# High-throughput in vivo phenotyping of chicken carcass traits based on digital radiographic imaging

**DOI:** 10.1186/s40104-026-01479-8

**Published:** 2026-08-04

**Authors:** Yidan Yan, Lei Wei, Yuan Zhou, Ruitao Tian, Yuhang Sun, Yuanling Zhang, Yang Liu, Pingtao Luo, Kun Yu, Yuzhe Wang, Hanyu Wu, Xiaoxiang Hu

**Affiliations:** 1 State Key Laboratory of Animal Biotech Breeding, College of Biological Sciences, China Agricultural University, Beijing, 100193 China; 2 Key Laboratory of White Feather Broiler Biological Breeding, Ministry of Agriculture and Rural Affairs, Fujian Sunnzer Biotechnology Development Co., Ltd., Guangze, 354100 China; 3 National Research Facility for Phenotypic and Genotypic Analysis of Model Animals, China Agricultural University, Beijing, 100193 China; 4 College of Animal Science and Technology, China Agricultural University, Beijing, 100193 China; 5 Frontiers Science Center for Molecular Design Breeding (MOE), China Agricultural University, Beijing, 100193 China

**Keywords:** Carcass traits, Digital radiography, High-throughput phenotyping, Non-destructive measurement, Poultry breeding, Phenomics

## Abstract

**Background:**

Accurate phenotyping of breast, drumstick, and wing yield is essential for genetic improvement in poultry breeding. However, these key economic traits can traditionally be measured only after slaughter, forcing breeding programs to rely on time-consuming, costly, and operator-biased sibling testing. This has become a major challenge for large-scale, high-precision, and non-destructive phenotypic evaluation in commercial poultry breeding. In this study, a non-destructive in vivo phenotyping framework based on digital radiography (DR) imaging combined with deep learning and machine learning methods is established.

**Results:**

A standardized DR image acquisition platform is constructed for in vivo phenotyping of live chickens. A lightweight multi-objective segmentation network, DRSegNet, is designed to achieve precise segmentation of breast, drumstick, and wing regions, with Dice coefficients higher than 97%. Morphological features were extracted from the segmentation masks and fed into machine learning models for weight regression. Five-fold cross-validation revealed strong predictive performance: the best-performing models achieved mean Pearson correlation coefficients of 0.8972, 0.7696, and 0.8049 for breast, drumstick, and wing weights, respectively. A phenotypic index (PI) and carcass balance index (CBI) are further constructed to enable comprehensive and balanced multi-trait selection. The deployed system supports a complete detection cycle of approximately 40 s per chicken, allowing high-throughput in vivo detection.

**Conclusions:**

This DR-based non-destructive framework enables reliable, real-time, and high-throughput in vivo prediction of key carcass traits in live chickens. It transforms traditional post-slaughter measurements into in vivo phenotypes obtainable directly from individual birds, may improve the accuracy and efficiency of breeding selection, and has the potential to shorten the breeding cycle. This study provides a practical and scalable technical solution for intelligent phenotyping in poultry, and may contribute to data-driven precision breeding systems.

**Supplementary Information:**

The online version contains supplementary material available at 10.1186/s40104-026-01479-8.

## Introduction

With the continuous growth of the global population and the stable development of the economy, the demand for animal protein worldwide is constantly increasing [1]. Poultry meat, particularly chicken, has become a major component of global meat consumption because of its relatively low production cost, high feed efficiency, and favorable nutritional value [2]. Within the chicken carcass, breast, leg, and wing represent the principal meat-yielding parts and account for a large proportion of carcass value. Therefore, their development and yield are directly relevant to breeding efforts aimed at improving the overall efficiency of meat production [3].

From a breeding perspective, the phenotypic measurement of carcass-related traits is of critical importance and has long been a focus of livestock breeding enterprises [4]. However, traits related to the breast, leg, and wing have long been recognized as typical post-mortem traits, which can usually only be measured after slaughtering. Therefore, in breeding practice, these traits are often indirectly evaluated through sibling testing to infer the genetic potential of candidate individuals for the same trait. This strategy is not only time-consuming, laborious, and costly, but also has inherent limitations. Because the sibling individuals used for phenotype determination need to be slaughtered, the phenotype information obtained is not completely consistent with the individuals actually retained for selection, inevitably introducing sampling bias and posing long-term challenges to large-scale and high-precision phenotype identification in poultry breeding [5].

In recent years, advances in imaging technology and computer vision have opened up new paths for non-destructive phenotype identification of animals, enabling indirect evaluation of body composition and growth related traits of living individuals [6, 7]. Multiple studies have explored the use of two-dimensional (2D) and three-dimensional (3D) imaging systems to predict body weight and tissue composition in livestock such as pigs, cows, and sheep [8, 9]. For instance, He et al. [10] used a 3D depth camera and an improved multi-object tracking model to predict morphological traits in pigs, while Pan et al. [11] applied a convolutional neural network (CNN)-based model to segment tissue regions in pig CT images and estimate the weight of fat, lean meat, and bone. Suwannakhun and Daungmala [12] combined digital image processing with deep learning for multi-feature weight estimation in pigs. Ruchay et al. [13] developed a depth-camera-based 3D vision system for accurate body measurement of live cattle, while Bai et al. [14] proposed a lightweight mobile model for real-time cattle weight estimation from ordinary photos. In sheep breeding, Zhang et al. [15] and Abdelhady et al. [16] employed image analysis techniques to extract body dimensions and predict weight. Studies in rabbits by Matics et al. [17] and Csóka et al. [18] also demonstrated the potential of image-based muscle segmentation and weight prediction. Similar efforts have been made in chicken research. Mortensen et al. [19] employed 3D cameras combined with image processing algorithms and Bayesian neural networks to estimate broiler chicken weight. De Aguiar et al. [20] utilized dual-energy X-ray absorptiometry (DEXA) to establish and validate regression equations for accurate prediction of broiler body composition. Sun et al. [21] constructed a classification and segmentation pipeline for CT slices of chicken drumsticks to realize screening of key slices and weight prediction.

Despite these advances, automatic measurement of internal traits in live poultry remains challenging. Most 2D image-based methods are limited to whole-body weight estimation and cannot differentiate among internal parts [22]. While 3D imaging techniques such as CT and MRI provide detailed anatomical information, they involve long scanning times, require anesthesia, and may induce stress in animals, which limit their use in high-throughput farming scenarios [23]. In addition, most phenotypic prediction methods based on 2D images are designed for single-trait analysis. To meet different phenotypic requirements, more data need to be processed for separate modeling and prediction [24]. This not only increases the workload but also generates more redundant data. On the other hand, due to the complexity of 3D data modeling, 3D-based phenotypic prediction methods typically require extensive data preprocessing and substantial computational resources, which pose additional challenges for practical field applications.

To address these challenges, this study presents a non-destructive framework for estimating weights of key chicken parts—breast, drumstick, and wing—using DR image. We introduce DRSegNet, a lightweight segmentation network that integrates a Multi-Scale Feature Extraction (MSFE) module with a Transformer layer to capture both local details and global context. The architecture employs three dedicated decoder branches, each enhanced with a Global Context and Local Attention (GCLA) module, to accurately segment the target regions. Following segmentation, morphological features are extracted from the predicted masks and fed into machine learning models for weight regression. This approach demonstrated high segmentation accuracy and reliable weight prediction. Beyond methodological development, this research aimed to create a practical system for breeding and production. The deployed software enables rapid, on-site phenotypic screening, addressing the need for real-time detection in breeding operations. By automating a traditionally manual and destructive process, the framework increases the level of automation in poultry phenotyping. Furthermore, the system can generate standardized, high-throughput phenotypic data, providing a data resource for downstream production optimization and forming a data-driven link to guide upstream genetic selection.

## Materials and methods

### Experimental data and DR imaging platform

**Fig. 1:**
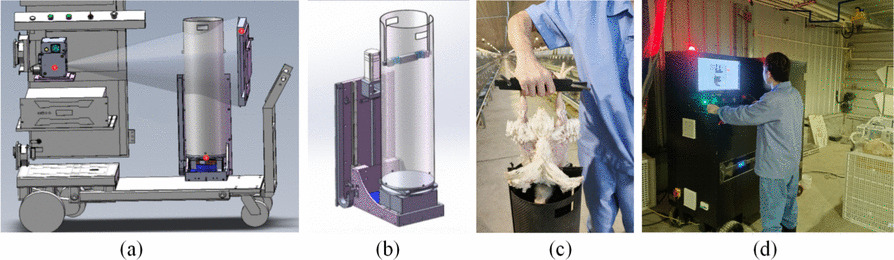
Mobile digital radiography (DR) imaging system for in vivo phenotyping of live chickens. **a** Overall structure of the mobile DR imaging system, including the X-ray source, detector, and control unit. **b** Customized sample chamber designed for stable positioning of live chickens during imaging. **c** Representative posture of a live chicken during in vivo DR image acquisition. **d** On-farm deployment of the DR system demonstrating high-throughput in vivo image acquisition under practical breeding conditions

All experimental samples were derived from Fujian Sunnzer Biotechnology Development Co., Ltd. (Guangze, China). The birds were white-feathered broilers with a defined genetic background, reared under standardized husbandry conditions until 39 d of age, covering the 36-, 37-, 38-, and 39-day-old cohorts. To minimize potential interference of gastrointestinal contents with imaging and carcass measurements, all birds were fasted for 12 h prior to data collection. A custom DR imaging system was integrated to construct an image acquisition platform for non-destructive imaging of live broilers (Fig. 1). The system consisted of a digital X-ray source and a flat-panel detector arranged in a fixed geometric configuration to acquire standardized DR images of each bird. During imaging, each bird was placed in a dedicated imaging chamber with mild restraint applied to the claws and wings to maintain a consistent posture and reduce motion artifacts. The chamber was then positioned in the custom DR acquisition platform, and individual DR images were captured for each bird under identical standardized settings. To ensure operator safety during DR imaging, systematic radiation protection measures were strictly implemented, the entire imaging system is enclosed within a lead-shielded structure to effectively reduce the risk of radiation leakage. The customized DR imaging system for automated animal phenotype extraction has undergone official radiation protection inspection before delivery, and fully complies with national standards, including GBZ 117–2015 Requirements for Radiological Protection in Industrial X-ray Detection and GB 18871–2002 Basic Standards for Protection Against Ionizing Radiation and for the Safety of Radiation Sources.

During operation, the system is equipped with fully enclosed shielding structures, protective doors and safety interlock devices, as well as clear radiation warning signs and operational indicators to enable timely protection responses under abnormal conditions. Meanwhile, multi-point monitoring of the ambient radiation dose rate was conducted at multiple locations around the equipment, including the operator’s working area and protection boundaries. The monitoring results confirmed that the radiation level at all detection points was far below the statutory limit.

Furthermore, standardized radiation safety protocols were strictly implemented throughout the experiment. Only trained and qualified personnel were allowed to operate the equipment, and unauthorized access to the scanning area was prohibited. Collectively, these multilayered safeguards ensured that operator radiation exposure remained within safe and compliant limits during all experimental procedures.

Post-imaging, DR images of each bird were automatically transmitted to a control terminal, assigned a unique sample ID, and stored for subsequent analysis. During image acquisition, imaging parameters were kept constant for all samples, and birds were imaged following a unified protocol to ensure inter-individual consistency and minimize technical variation. Acquired DR images were set to a resolution of 1,536 × 1,536 pixels and stored in a lossless format for downstream analysis. Individual samples exhibiting excessive motion artifacts or poor image quality were discarded and excluded from subsequent analyses. Following DR imaging, all birds were humanely euthanized following standard procedures, and slaughtered to collect ground truth data. Trained technicians dissected the birds to separate the pectoralis major and minor (collectively referred to as breast), drumstick, and wing tissues. The weight of each tissue component was measured using an electronic balance and recorded; these measured values served as the reference phenotypic ground truth for model training and evaluation.

### Image preprocessing and annotation

Unified preprocessing was performed on all raw DR images prior to model training and analysis. The original DR image has a high spatial resolution and relatively low overall gray intensity. Using it directly for model training would increase computational complexity and memory consumption. Thus, a standardized DR image preprocessing pipeline was developed, primarily including image enhancement and normalization operations, to improve the overall contrast and visibility of anatomical structures. All preprocessing steps were uniformly applied to the entire dataset to avoid introducing additional technical bias. For supervised training of the segmentation network, reference masks were manually annotated using the LabelMe tool [25]. All image annotations in this study were jointly performed by two professional annotators with a background in computational biology and experienced anatomical technicians from the breeding farm, ensuring accurate identification of anatomical structures from both technical and practical perspectives. Given the distinct structural features of the breast, drumstick, and wing regions in DR images, annotation difficulty was relatively low, and most regions were labeled consistently. To minimize inter-annotator variation and verify annotation consistency, any inconsistent regions were thoroughly reviewed and confirmed by a third senior annotator until a unified consensus was reached. This rigorous procedure ensured annotation consistency and reliability. All acquired DR images and their matched masks constituted the dataset for model training and evaluation, and also provided a foundation for the subsequent design of the network architecture and performance analysis.

### Network architecture

**Fig. 2:**
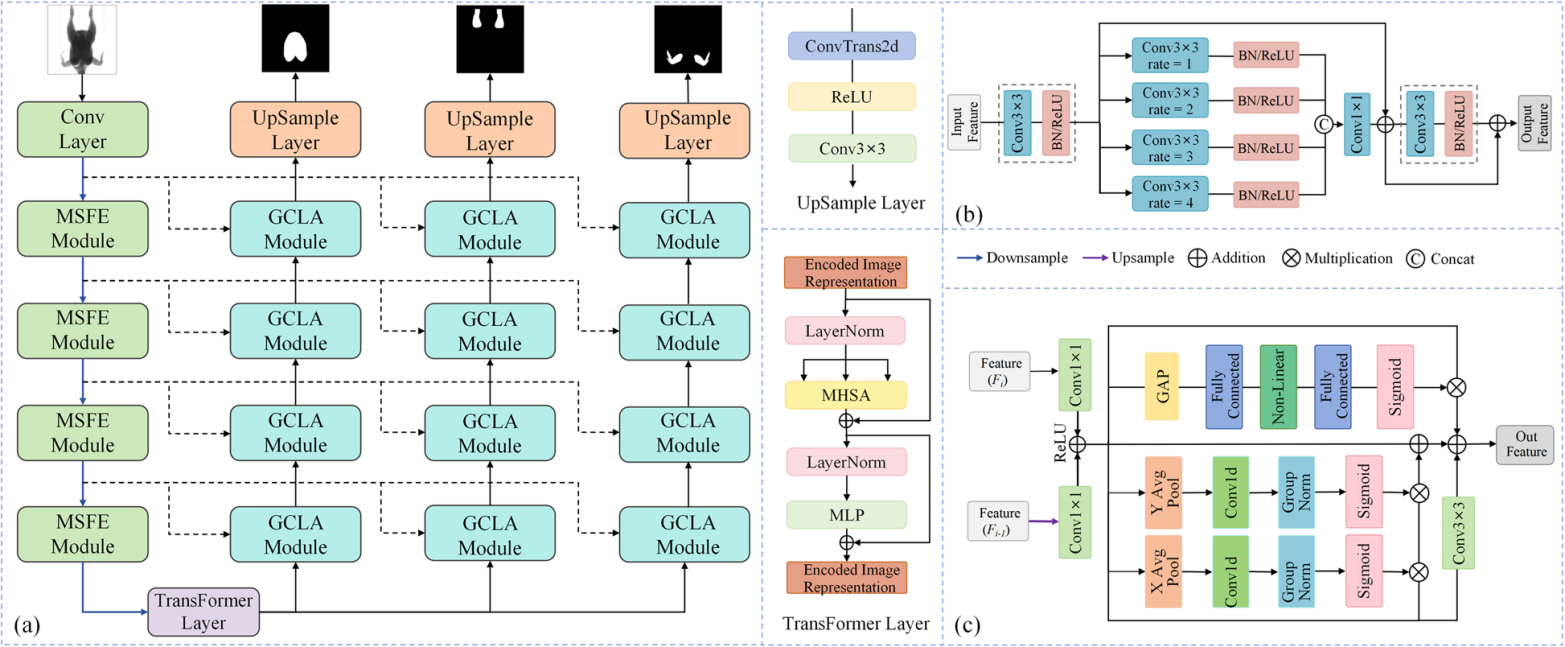
Architecture of the proposed DRSegNet structure. **a** Overall architecture of the proposed DRSegNet. **b** Structure of the multi-scale feature extraction (MSFE) module. **c** Structure of the global context and local attention (GCLA) module

To achieve accurate segmentation of multiple key target regions from DR images and improve data utilization efficiency, a lightweight multi-target segmentation network named DRSegNet was developed in this study (Fig. 2). The network adopted a traditional encoder-decoder architecture, with three independent decoders designed to optimize different segmentation targets, respectively. The encoder was responsible for feature extraction from input DR images. To capture anatomical structures at multiple scales, the encoder incorporated a proposed multi-scale feature extraction module for hierarchical feature extraction across different scales. In addition, a Transformer network was introduced to model long-range dependencies and global contextual information, which facilitated the network to identify and distinguish overlapping or adjacent regions in DR images. The decoder was configured with three segmentation branches for distinct targets, corresponding to the breast, drumstick, and wing regions, respectively. Each branch integrated global contextual channels and local information to optimize fine-grained characterization of local regions and enhance key channel information, effectively reducing interference between different anatomical regions. This multi-branch design enabled the network to segment multiple traits simultaneously in a single forward pass, providing a robust foundation for downstream phenotypic trait estimation and allowing targeted optimization of segmentation targets.

A schematic diagram of the DRSegNet architecture is presented in Fig. 2, and detailed descriptions of each module are provided in the following subsections.

#### Multi-scale feature extraction module

Multi-scale feature learning is crucial in the field of computer vision, especially in image segmentation tasks, as it enables models to capture patterns of different scales and accurately identify targets of different sizes [26]. To enhance the ability to capture multi-scale features, we propose an MSFE module by combining multi-scale feature learning with a residual connection structure.

The input feature *F*_*in*_ is first projected through a 3 × 3 convolution followed by a batch normalization (BN) and ReLU layer to generate feature *F*_1_. Subsequently, the feature *F*_1_ is divided into four equal parts along the channel dimension, and each part is processed in parallel through a 3 × 3 convolutional layer with dilation rates of 1, 2, 3 and 4. This design enables the model to efficiently capture multi-scale contextual information. The result features of parallel branches are concatenated and fused through a 1 × 1 convolutional layer. Residual connection combines the fused output with *F*_1_ to generate feature *F*_2_. The final *F*_2_ is refined by another 3 × 3 convolution, and the result is added to *F*_2_ through a second residual connection to obtain the final output feature *F*_*out*_. The corresponding calculations are shown in Eqs. (1)–(4):

1 $${F}_{1}=ReLU(BN\left(\left({Conv}_{3\times 3}\left({F}_{in}\right)\right)\right))$$

2 $${F}^{\prime}_{i}=ReLU\left(BN\left({Conv}_{3\times 3}^{d=i}\left({F}_{1-i}\right)\right)\right),i=\mathrm{1,2},\mathrm{3,4}$$

3 $${F}_{2}={F}_{1}+{Conv}_{1\times 1}(Cat\left({F}_{1}^{\prime},{F}_{2}^{\prime},{F}_{3}^{\prime},{F}_{4}^{\prime}\right))$$

4 $${F}_{out}={F}_{2}+ReLU(BN\left({Conv}_{3\times 3}\left({F}_{2}\right)\right))$$

#### Transformer layer

In image segmentation tasks, classical CNNs are limited by the size of local receptive fields, making it difficult to effectively model long-distance pixel dependencies. In contrast, Transformer [27] utilizes multi-head self-attention (MHSA) mechanism to capture cross-regional contextual information of images and achieve global feature interaction, enhancing its ability to model complex semantic relationships. Therefore, this study integrates Transformer into multi-target segmentation tasks, establishing feature associations based on global context, and passing these features to each decoder. This method not only compensates for the inherent shortcomings of CNN in global information modeling, but also enhances the semantic understanding ability of each decoder channel.

As illustrated in the network architecture shown in Fig. 2a, the input features are first transformed into their encoded representations via operations such as embedding to yield the feature map *F*_*E*_. Subsequently, layer normalization (LN) is applied to the input features, which are then fed into the MHSA mechanism followed by a residual connection to generate the feature map *F*_*M*_. Next, LN is performed on *F*_*M*_, and the resulting features are processed through a multi-layer perceptron (MLP) with another residual connection. Finally, the features are reshaped to the dimension of the original input features and output as the final result *F*_*out*_. The corresponding calculation formula is given as follows:

5 $${F}_{M}=MHSA\left(LN\left({F}_{E}\right)\right)+{F}_{E}$$

6 $${F}_{out}=MLP\left(LN\left({F}_{M}\right)\right)+{F}_{M}$$

#### Global context and local attention module

In image segmentation tasks, features extracted by the encoder do not always contribute effectively to target region segmentation. Although deeper-layer features provide richer semantic information, they may also contain features from irrelevant background regions. To strengthen the network’s focus on valid regional features, we propose a GCLA module. This module performs channel-wise feature compression and key feature activation using contextual features from the encoder-decoder, while enhancing the learning capability for local boundary regions, thus improving the overall representational capacity of each decoder network for key segmentation regions.

The GCLA module takes features from the encoder (*F*_*i*_) and decoder (*F*_*i−*1_) as inputs. First, the decoder features are upsampled to adjust their spatial dimensions. These two feature components are then processed separately through a 1 × 1 convolutional layer and activated by ReLU to generate fused features *F*_*in*_. Subsequently, *F*_*in*_ is fed into two parallel branches: a channel attention branch implementing the squeeze-and-excitation (SE) mechanism [28], and a local attention branch adopting the efficient local attention (ELA) architecture [29]. The computational procedure is defined as follows:

7 $${F}_{in}=ReLU({Conv}_{1\times 1}\left(Up\left({F}_{i-1}\right)\right)+{Conv}_{1\times 1}\left({F}_{i}\right))$$

In the feature squeeze and excitation channel, the input features are scaled through global average pooling (GAP), then undergo feature transformation via a fully connected network layer, and finally are multiplied by the original input features after passing through a sigmoid activation function. This realizes the compression and activation of feature information in the channel dimension. The corresponding calculation is as follows:

8 $${F}_{out1}={F}_{in}\times Sigmoid(MLP\left(GAP\left({F}_{in}\right)\right))$$

In the local attention channel, the input features undergo pooling operations along the *X*-axis and *Y*-axis respectively, then pass through a 1D convolutional layer. After that, the two parts of features are processed by group normalization (GN) and sigmoid activation function layers respectively to obtain feature activation coefficients *a*_*X*_ and *a*_*Y*_ in two different dimensions. Finally, these coefficients are multiplied by the input features to generate the local attention feature map. The corresponding formulas are as follows:

9 $${a}_{X}=Sigmoid(GN(Conv1d\left({P}_{X}\left({F}_{in}\right)\right)))$$

10 $${a}_{Y}=Sigmoid(GN\left(Conv1d\left({P}_{Y}\left({F}_{in}\right)\right)\right))$$

11 $${F}_{out2}={F}_{in}\times {a}_{X}\times {a}_{Y}$$

The outputs from both pathways, the original input *F*_*in*_ and a transformed feature from a 3 × 3 convolution are aggregated to produce the final enhanced output. This module realizes the integration of contextual information, ensuring that the network can compress and activate features and capture target attention, while reducing feature loss and retaining the original effective features. The corresponding calculation is as follows:

12 $${F}_{out}={F}_{out1}+{F}_{out2}+{F}_{in}+{Conv}_{3\times 3}({F}_{in})$$

### Machine learning-based weight prediction

Following the segmentation of breast, drumstick, and wing regions by DRSegNet, a set of morphological features was extracted from each predicted segmentation mask to quantitatively characterize the size and shape of the corresponding anatomical region. The complete list and description of all features are provided in Table 1. These features collectively capture both the regional morphological information derived from DR images and the global body size of each individual, forming a multi-dimensional feature matrix for subsequent regression analysis.

**Table 1:** Detailed description list of different features

No.	Feature	Description	Symbol
1	Pixel area	Number of pixels in the mask area	Pa
2	Convex area	Minimum number of pixels in the convex hull area	Ca
3	Perimeter	The number of pixels in the boundary area of the mask	P
4	Major axis length	The major axis length of the ellipse outside the mask area	Mal
5	Minor axis length	The short axis length of the ellipse outside the mask area	Mil
6	Upright width	The width of circumscribed upright rectangle	Uw
7	Upright height	The height of circumscribed upright rectangle	Uh
8	Minimum rectangle width	The width of minimum circumscribed rectangle	Mw
9	Minimum rectangle height	The height of minimum circumscribed rectangle	Mh
10	Outer circle diameter	Diameter of circumscribed circle	O
11	Live weight	Weight of chickens	Lw

Based on the constructed feature matrix, six commonly used machine learning regression models were employed to predict the weight of each carcass component (breast, drumstick, and wing): Random Forest, Extreme Gradient Boosting (XGBoost), Least Absolute Shrinkage and Selection Operator (Lasso) Regression, Bayesian Ridge Regression, Linear Regression, and Elastic Net. Each model takes the extracted morphological features as input and outputs the predicted weight of the corresponding body part. Model performance was evaluated using four metrics: Pearson correlation coefficient (PCC), root mean square error (RMSE), mean absolute error (MAE), and mean relative error (MRE).

### Calculate the comprehensive phenotype value

To achieve efficient screening of multiple traits such as breast, drumstick, and wing weights of chickens, and to avoid the problems of imbalance in carcass proportions and decline in comprehensive production performance that may be caused by single-trait selection, this study proposes a multi-trait comprehensive evaluation method based on Z-score normalization, aiming to provide quantitative evaluation indicators that combine overall advantages and proportion balance for agricultural practices. By calculating the phenotypic index (PI) and carcass balance index (CBI), a dual quantitative evaluation of individual phenotypic performance was achieved, providing a scientific basis for selecting ideal breeding individuals.

#### Phenotypic index

To comprehensively evaluate the overall performance of individuals in the three traits (breast, drumstick, and wing), the PI was constructed. By conducting weighted summation of the Z-scores of each trait, the quantitative ranking of multi-trait comprehensive performance was realized, thus enabling the selection of individuals with excellent comprehensive performance.

First, Z-score normalization was performed on the prediction results of each trait to convert the original trait values into relative values on a unified scale. Subsequently, weighted summation of the Z-scores of each trait was carried out to achieve quantitative ranking of multi-trait comprehensive performance, thereby screening out individuals with outstanding comprehensive performance. The calculation formula is shown as follows:

13 $${Z}_{t,i}=\frac{{x}_{t,i}-{\mu}_{t}}{{\sigma}_{t}}$$

where *Z*_*t*,*i*_ denotes the Z-score standardized value of the *t*-th trait (breast/drumstick/wing) for the *i*-th individual (corresponding to *Z*_*B*_ , *Z*_*D*_ , *Z*_*W*_); *x*_*t,i*_ represents the original predicted value of the *t*-th trait for the *i*-th individual; *μ*_*t*_ denotes the mean of the original values of the *t*-th trait across all individuals in the experimental population; ${\sigma}_{t}$ means the standard deviation of the original values of the *t*-th trait across all individuals in the experimental population.

It is worth noting that Z-score standardization only harmonizes the numerical scales of different carcass traits and serves as a necessary preprocessing step independent of trait weighting. The sign of the Z-score reflects whether the individual's trait performance is superior or inferior to the population mean, while the absolute value indicates the degree of deviation from the mean. This standardization lays a unified data foundation for the subsequent calculation of PI and CBI.

14 $${PI}_{i}={w}_{1}\times {Z}_{B}+{w}_{2}\times {Z}_{D}+{w}_{3}\times {Z}_{W}$$

where *w*_*t* _denotes the weight coefficient of the *t*-th trait. Equal weights are used in this study, where weights can be adjusted based on breeding objectives and the economic value of traits.

A higher PI value indicates more excellent comprehensive performance of an individual in the three traits. By sorting the PI values in descending order, individuals with top-ranked comprehensive performance in the population (e.g., the top 10 samples) can be rapidly selected.

#### Carcass balance index

To carry out breeding selection in a multi-faceted manner and avoid the problem of unbalanced carcass proportions characterized by "outstanding performance in a single trait with lagging performance in other traits" in individuals with high PI values, the construction of a CBI was proposed. By calculating the standard deviation of the Z-scores of an individual’s three traits, the degree of dispersion of the relative performance of each trait was quantified, which reflects the balance of carcass proportions. The calculation formula is shown as follows:

15 $${CBI}_{i}=SD({Z}_{B},{Z}_{D},{Z}_{W})$$

where *SD* represents the standard deviation function, used to reflect the dispersion of the three Z-score values.

A smaller CBI value indicates a smaller difference in the Z-scores of an individual’s three traits and a more balanced carcass proportion. In contrast, a larger CBI value indicates obvious imbalance in trait performance, which fails to meet the breeding goal of coordinated development. The CBI was designed as an auxiliary screening constraint rather than a primary target trait for directional selection. It will not be used alone to conduct individual selection; instead, it serves as an additional filtering threshold to exclude individuals with severely unbalanced carcass composition.

## Results

### DR image acquisition system for in vivo phenotyping

To support non-destructive phenotyping of carcass-related traits in live poultry, a mobile DR image acquisition system was established to enable standardized and efficient collection of in vivo imaging data. The sample chamber used for sampling is made of carbon fiber material, which ensures high X-ray transmittance and compatibility with routine sterilization, thereby meeting biosafety requirements.

In order to maintain imaging stability, we slightly fixed the chicken claw area with constraint rods, and uniformly fixed the chicken wing area with wing clips. Then, we placed it in the sample chamber for uniform posture fixation (Fig. 1b and c), which kept the body positions of all sampled broilers consistent. By taking advantage of the natural tendency of chickens to remain calm when upside-down, DR images can be obtained from conscious chickens without the use of anesthesia, thereby minimizing handling stress and facilitating rapid phenotypic measurements.

The sampling system built in this study implements a standardized workflow for DR image acquisition and data storage, ensuring consistency in the localization and imaging conditions of all samples. Under normal operating conditions, the average image acquisition time for each broiler is about 8 s, fully supporting breeding applications such as high-throughput sample collection and phenotype analysis in actual farm environments.

Overall, the DR imaging platform established in this study can effectively acquire stable, high-quality, and reproducible DR images of poultry, while providing a reliable data foundation for subsequent image segmentation and accurate assessment of key phenotypic traits.

### Dataset collection and processing

**Fig. 3:**
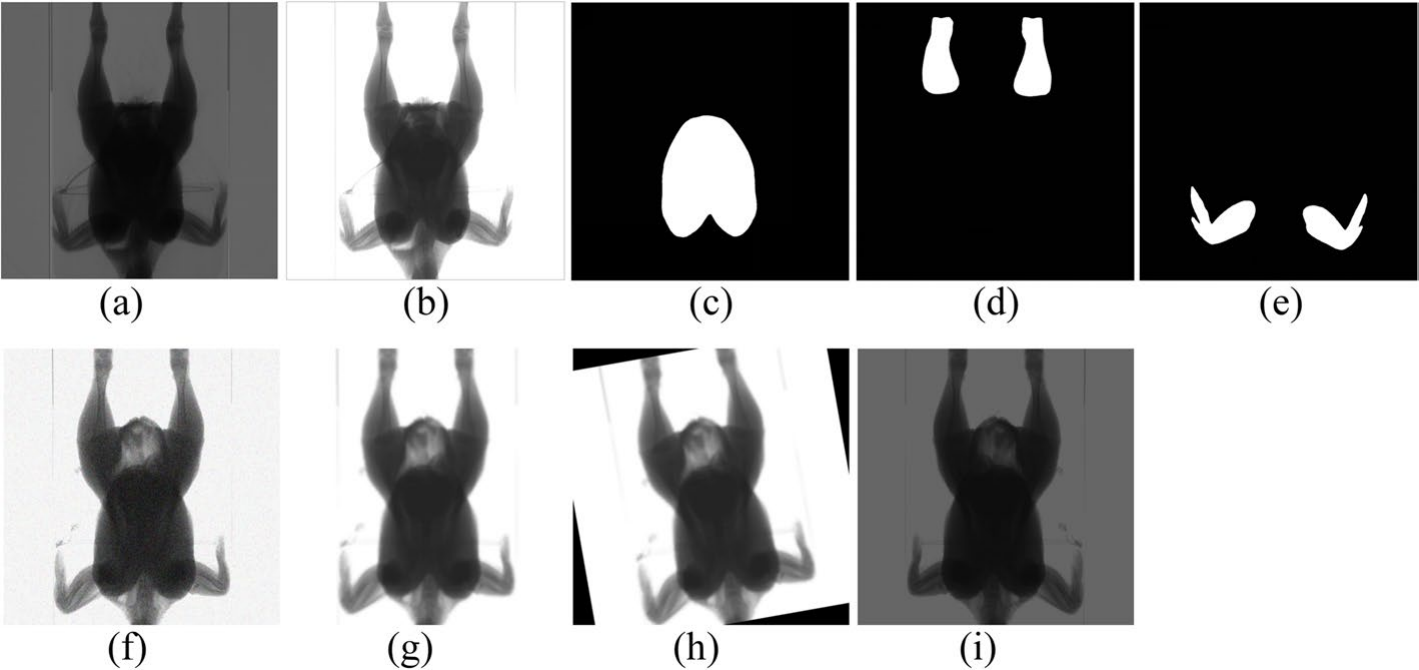
Representative samples from the in vivo poultry DR image dataset. **a** Original DR image. **b** DR image after image enhancement. **c**–**e** Manual annotations of the breast, drumstick, and wing regions. **f**–**i** Augmented images generated through noise perturbation, blurring, rotational blurring, and horizontal flipping

To establish a high-throughput in vivo evaluation system for poultry, a collaborative study was conducted with a commercial poultry farm in this research. A total of 502 white-feathered broiler samples were initially collected for DR image acquisition using the constructed image acquisition platform. However, 12 samples were excluded due to excessive motion during the imaging process, accounting for approximately 2.39% of the total collected samples. Finally, valid DR images of 490 white-feathered broilers were successfully captured (Fig. 3a), with each valid sample assigned a unique sample ID for convenient identification and subsequent data tracking. The raw images exhibited relatively large file sizes and low brightness, which would increase recognition difficulty, memory usage and training time if used directly. Therefore, all images were first enhanced via histogram-based processing to improve contrast and clarity, and then uniformly resized to 512 × 512 pixels (Fig. 3b). Subsequently, professional annotators manually labeled the breast, drumstick and wing regions on the uniformly processed images (Fig. 3c–e).

The dataset was randomly divided into training and testing subsets at an 8:2 ratio, resulting in 392 training images and 98 test images. To increase data diversity and improve model robustness, data augmentation strategies were applied to datasets, including noise perturbation, blurring, rotational transformations, and horizontal flipping (Fig. 3f–i).

Overall, this dataset provides comprehensive coverage of major carcass regions in live chickens and serves as a robust basis for subsequent segmentation performance evaluation and imaging-based carcass trait quantification.

### Multi-target segmentation performance evaluation

The automated segmentation performance of key carcass anatomical parts, including the breast, drumstick, and wing, was evaluated using the proposed DRSegNet model. Segmentation accuracy was assessed through qualitative visual comparison and quantitative evaluation against multiple representative baseline segmentation models, including UNet [30], SegNet [31], AttentionUNet [32], TransUNet [33], CANet [34], MSUNet [35], ConvUNeXt [36], ASENet [37] and DCSAUNet [38].

**Fig. 4:**
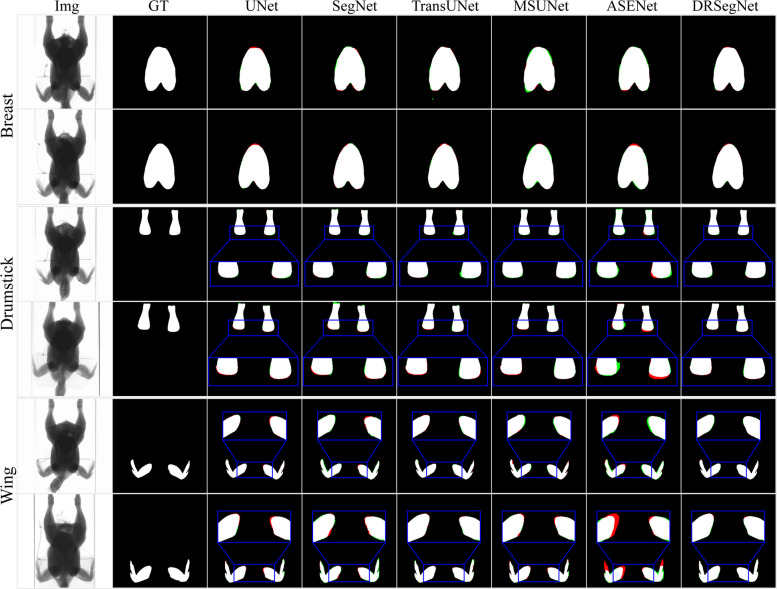
Visual comparison of segmentation results for breast, drumstick, and wing regions across different models. For each anatomical region, the first column shows the original DR image, and the second column shows the corresponding ground truth (GT). The remaining columns present segmentation results generated by different models. White areas indicate correctly segmented regions, red areas indicate under-segmentation, and green areas indicate over-segmentation. Blue boxes highlight representative local regions with complex boundaries or overlapping structures, which are enlarged to facilitate detailed comparison

Representative visual comparisons of segmentation results are shown in Fig. 4. For breast segmentation, most models were able to identify the main anatomical area. However, clear differences were observed at anatomically complex boundaries, particularly around the keel region. Several baseline models exhibited under-segmentation or irregular contours in these areas, whereas DRSegNet generated more complete and smoothly aligned boundaries that closely matched the reference annotations.

In drumstick segmentation, minor boundary inaccuracies were frequently observed at joint intersections for several baseline models, leading to local deviations in contour delineation. In contrast, DRSegNet achieved the best consistency with the ground truth masks in terms of overall shape and fine-grained local structural details of each region. The accurate segmentation of the wing region is challenging due to its overlap with the breast region. However, it is evident from the figures that compared with all baseline segmentation results, the proposed DRSegNet in this study achieves the highest Intersection over Union (IoU) and Dice scores among all evaluated methods. It not only accurately segments the edges of the wing tips but also precisely delineates the overlapping areas, outperforming the other comparative methods evaluated here in terms of completeness and boundary refinement of the segmented regions.

The quantitative evaluation results (Table 2) further validate the visual segmentation performance and support the favorable performance of the proposed DRSegNet. Across the three anatomical regions, DRSegNet consistently achieved the highest scores in core metrics (Precision, Recall, IoU, Dice coefficient), with its IoU and Dice values remaining the optimal across all tasks.

**Table 2:** Performance comparison of different segmentation models for multi-target segmentation on key chicken regions

Tissues	Model	Precision	Recall	IoU	Dice
**Breast**	UNet	97.62	98.03	95.74	97.82
	SegNet	97.59	98.28	95.95	97.93
	AttentionUNet	98.03	98.33	96.42	98.17
	TransUNet	97.79	98.16	96.03	97.96
	CANet	98.32	98.11	96.48	98.21
	MSUNet	96.98	97.51	94.60	97.21
	ConvUNeXt	98.95	97.57	96.56	98.24
	ASENet	97.78	98.07	95.92	97.91
	DCSAUnet	97.90	98.58	96.52	98.22
	DRSegNet	99.05	98.68	97.75	98.86
**Drumstick**	UNet	98.42	98.17	96.79	98.29
	SegNet	97.40	98.29	95.91	97.84
	AttentionUNet	98.34	98.33	97.16	98.33
	TransUNet	98.57	98.01	96.77	98.28
	CANet	98.58	98.16	96.94	98.37
	MSUNet	98.63	97.41	96.25	98.01
	ConvUNeXt	98.14	98.48	96.82	98.31
	ASENet	92.74	96.96	90.18	94.75
	DCSAUnet	98.36	97.82	96.40	98.08
	DRSegNet	98.67	98.68	97.53	98.67
**Wing**	UNet	95.85	96.52	92.84	96.15
	SegNet	94.67	96.81	91.99	95.69
	AttentionUNet	96.27	97.22	93.91	96.72
	TransUNet	97.08	96.57	94.04	96.80
	CANet	95.09	97.06	92.66	96.02
	MSUNet	96.52	96.28	93.25	96.38
	ConvUNeXt	97.06	97.07	94.51	97.05
	ASENet	90.49	92.72	84.56	91.47
	DCSAUnet	96.93	97.06	94.38	96.98
	DRSegNet	97.52	97.36	95.22	97.43

Precision, Recall, Intersection over Union (IoU), and Dice coefficient were used to evaluate segmentation accuracy. Higher values of Precision, Recall, IoU, and Dice indicate better segmentation performance

In summary, DRSegNet demonstrated stable and robust performance in the multi-target segmentation of key carcass anatomical regions in live broilers. Its consistently leading performance across all three regions, especially the challenging wing region, provides accurate and reliable segmentation results for downstream phenotypic estimation.

The superior segmentation performance of DRSegNet can be attributed to its tailored architectural and algorithmic advantages for poultry DR imaging. First, the MSFE module captures multi-scale anatomical patterns with different dilation rates, enabling the network to adapt to the large size variations of breast, drumstick, and wing regions while preserving fine structural details that are easily lost in conventional convolutions. Second, the integration of a Transformer layer compensates for the limited receptive field of CNNs, modeling long-range spatial dependencies to help distinguish overlapping or adjacent structures (e.g., wing overlapping with breast) that confuse traditional segmentation models. Third, the GCLA module embedded in each decoder branch strengthens channel-wise importance and local boundary awareness simultaneously, suppressing background noise and sharpening edges around joints and keel regions where most baseline models produce under-segmentation or irregular contours. Furthermore, the three-branch parallel decoder design allows independent optimization for each target region, reducing mutual interference between anatomical parts and improving precision for small or complex structures such as the wing. Together, these customized components enable DRSegNet to achieve higher precision, recall, IoU, and Dice scores than other advanced methods, especially in challenging scenarios involving overlapping tissues, blurred boundaries, and structural complexity in broiler DR images.

### Ablation analysis of segmentation performance

To verify the impact of each component in the network on segmentation performance, we conducted ablation experiments. Table 3 summarizes the quantitative results of each network variant. Segmentation experiments on the breast, drumstick, and wing anatomical regions showed that removing a single module reduced segmentation performance. Compared with the complete DRSegNet integrating all modules, deleting either the MSFE module (Network 1) or the GCLA module (Network 2) led to decreased IoU and Dice coefficients. The most substantial decline in segmentation performance was observed when both modules were removed simultaneously (Network 3).

**Table 3:** Results of ablation experiments evaluating the contribution of individual modules in DRSegNet for multi-target segmentation

Methods	Chicken breast		Chicken drumstick		Chicken wing	
	IoU	Dice	IoU	Dice	IoU	Dice
Network1	97.58	98.77	97.36	98.58	94.84	97.23
Network2	97.63	98.79	97.32	98.42	94.77	97.19
Network3	97.31	98.34	97.12	98.27	94.18	97.02
DRSegNet	97.75	98.86	97.53	98.67	95.22	97.43

In the anatomically complex wing region, the fine structural details and overlap with the adjacent breast made segmentation particularly challenging, leading to notably pronounced differences in the segmentation results. Compared with the complete model, the simplified models with different modules removed exhibited lower IoU and Dice values for wing segmentation, further verifying the effectiveness of each module in the segmentation task.

Overall, DRSegNet incorporating all modules achieved the highest segmentation performance across all anatomical regions of the carcass.

### Multi-trait weight prediction based on machine learning

**Fig. 5:**
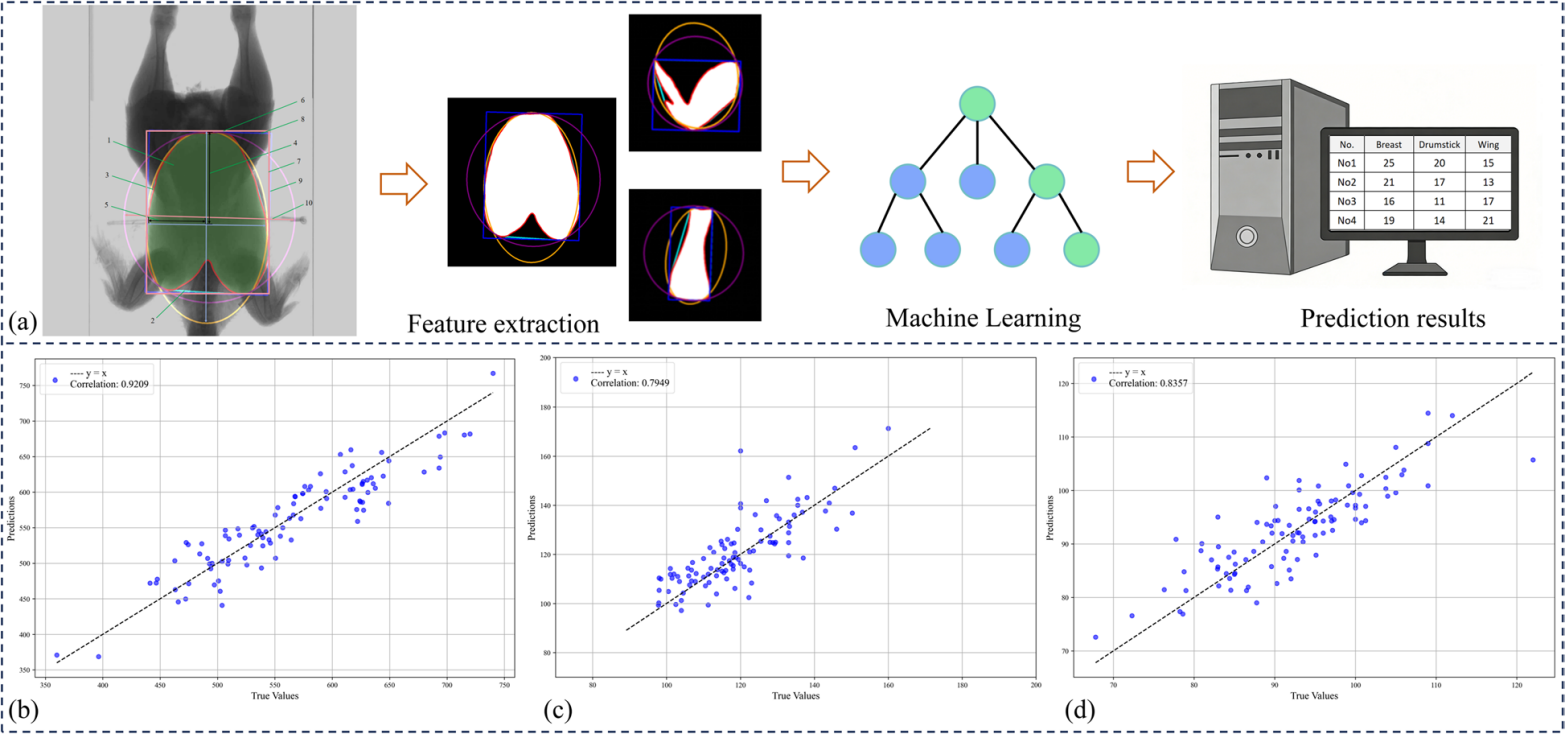
Workflow of morphological feature-based weight prediction and model fitting performance for key carcass-related traits. **a** Schematic illustration of the weight prediction pipeline, including feature extraction from segmented carcass components, machine learning-based regression modeling, and generation of predicted trait values. **b** Scatter plot of predicted versus measured breast weight. **c** Scatter plot of predicted versus measured drumstick weight. **d** Scatter plot of predicted versus measured wing weight. The dashed line represents the line of identity (*y* = *x*)

To accurately predict the weights of multiple chicken parts, such as breast, drumstick, and wing, key morphological features were extracted from each segmented region. Based on this analysis, a multi-dimensional feature matrix was constructed to characterize the size and shape of each part. Subsequently, a set of machine learning regression models, including Random Forest, XGBoost, Lasso, Bayesian Ridge, Linear Regression, and Elastic Net, were trained using these multi-scale features to directly predict the weight of each specific part from DR images (Fig. 5a).

As shown in Table 4, we conducted experiments with five-fold cross-validation. The tabulated values are averaged across all folds, and all models demonstrate different predictive capabilities for carcass component weights. For breast weight prediction, the Linear Regression model achieved the highest PCC (0.8972) and lowest RMSE (29.83 g) and MAE (23.57 g), closely followed by Bayesian Ridge and Lasso regression. For drumstick weight prediction, Random Forest outperformed other models, with PCC values of 0.7696, RMSE values of 12.33 g and MAE values of 7.93 g. For wing weight prediction, Lasso yielded the highest PCC value (0.8049), with comparable RMSE, MAE, and MRE across most models. The scatter plots in Fig. 5b–d show the prediction results from a single fold of the five-fold cross-validation.

**Table 4:** Predictive performance of different regression models for multi-objective prediction of key carcass-related trait weights

Trait	Model	PCC	RMSE, g	MAE, g	MRE, %
**Breast**	Random Forest	0.8630	34.14	27.06	4.81
	XGBoost	0.8591	34.69	27.47	4.87
	Lasso	0.8898	30.83	24.83	4.43
	Bayesian Ridge	0.8950	30.13	24.05	4.28
	Linear	0.8972	29.83	23.57	4.20
	Elastic Net	0.8804	32.67	25.91	4.64
**Drumstick**	Random Forest	0.7696	12.33	7.93	6.24
	XGBoost	0.7468	12.89	8.33	6.53
	Lasso	0.7024	13.72	8.89	6.89
	Bayesian Ridge	0.7070	13.62	9.01	7.02
	Linear	0.7008	13.76	9.23	7.22
	Elastic Net	0.6810	14.34	8.97	6.93
**Wing**	Random Forest	0.7680	5.78	4.53	4.92
	XGBoost	0.7823	5.61	4.46	4.84
	Lasso	0.8049	5.42	4.23	4.60
	Bayesian Ridge	0.8028	5.36	4.20	4.56
	Linear	0.8016	5.37	4.22	4.58
	Elastic Net	0.8048	6.00	4.73	5.17

PCC denotes the Pearson correlation coefficient between predicted and measured weights. RMSE and MAE represent the root mean square error and mean absolute error, respectively. MRE indicates the mean relative error. Higher PCC values and lower RMSE, MAE, and MRE values indicate better predictive performance

To evaluate whether the observed discrepancies in predictive performance across different models were statistically significant, we conducted pairwise non-parametric tests, and all analytical results are illustrated in Fig. S1(a–d). The −log_10_(*P*) heatmap (Fig. S1a) reveals that a large proportion of model pairs showed significant differences for breast prediction. For drumstick and wing weight prediction, only a limited subset of pairwise comparisons reached significance, while most comparisons were non-significant. The bar chart of median absolute errors (Fig. S1b) indicates only marginal disparities in the central tendency of prediction errors among models, while the boxplots of absolute prediction errors (Fig. S1c) clearly demonstrate extensive overlaps in error distributions across all models. Figure S1d presents heatmaps of four quantitative evaluation metrics for all regression models, including the PCC, RMSE, MAE, and MRE, which visually summarize the overall predictive performance of the six regression models on the weight prediction tasks of broiler breast, drumstick, and wing.

### Development and application of the non-destructive prediction model for multi-trait selection in chicken breeding

**Fig. 6:**
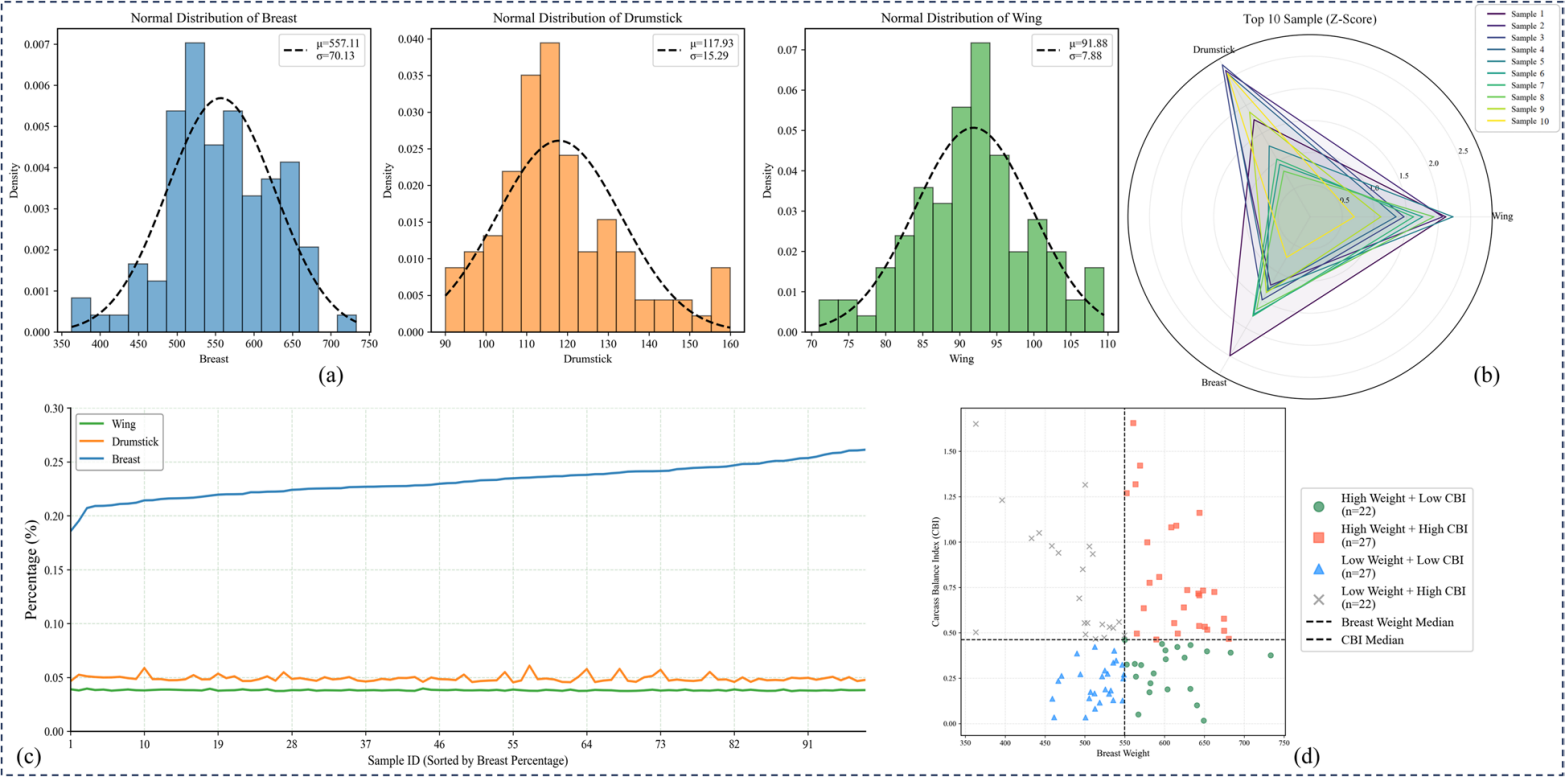
Multi-dimensional comprehensive assessment results of carcass traits of live poultry based on DR imaging. **a** Normal distribution map of predicted results for each region. **b** Radar chart of regional indices for the top 10 samples. **c** Comparison curve of muscle percentage calculated based on the predicted results of each region (samples sorted in ascending order according to breast muscle percentage). **d** Scatter plot of carcass balance index (CBI) versus breast weight (categorized by individual type)

The above experimental results demonstrate the effectiveness of the DR imaging-based prediction model proposed in this study for estimating the breast, drumstick, and wing weights of live chickens. Further analysis of the distribution of the predicted values for the test population shows that the distribution is as depicted in Fig. 6a. This pattern is similar to the distribution of quantitative traits, supporting the reliability of predicted results for breeding analysis with large batches of samples.

In order to transform the predicted weight of each key body into evaluation criteria that can be used in breeding practice, this study further constructed two quantitative indicators, PI and CBI, to form a multidimensional breeding evaluation system, providing a quantitative basis for screening excellent individuals with comprehensive performance and coordinated carcass structure.

Based on these phenotypic results, we propose calculating the PI using Z-score standardization to enable directional selection based on PI values. The top 10 individuals ranked by PI are shown in Fig. 6b. All of these top individuals maintain Z-scores above the population average for breast, drumstick and wing simultaneously, which means all three carcass traits exceed the population mean. Although minor differences exist in the relative superiority of each trait among samples and some individuals display a more prominent advantage in breast weight, the PI still enables the rapid screening of individuals with generally high comprehensive breeding value.

We then calculated and statistically analyzed the muscle percentages from all computed phenotypic results, and the correlation analysis results are presented in Fig. 6c. It can be observed that the correlation between breast and drumstick proportions is weak, suggesting that they may serve as relatively independent phenotypic selection targets. Wing percentage shows high stability, and wing weight itself shows a strong positive correlation with body weight. Thus, wing traits can be indirectly selected based on body weight. Additionally, the CBI was calculated based on the standardized trait values (Fig. 6d), which acts as a constraint during breast meat selection, helping to identify individuals with high breast weight and low structural imbalance, thereby improving yield while maintaining carcass balance.

Overall, the proposed DR-based prediction model enables simultaneous and non-destructive estimation of multiple carcass traits in live chickens. The combined use of PI and CBI supports balanced multi-trait selection rather than single-trait optimization. This strategy provides a practical tool for breeding high-quality broilers with balanced carcass development, in line with current poultry breeding goals.

### Runtime performance and end-to-end workflow evaluation

**Fig. 7:**
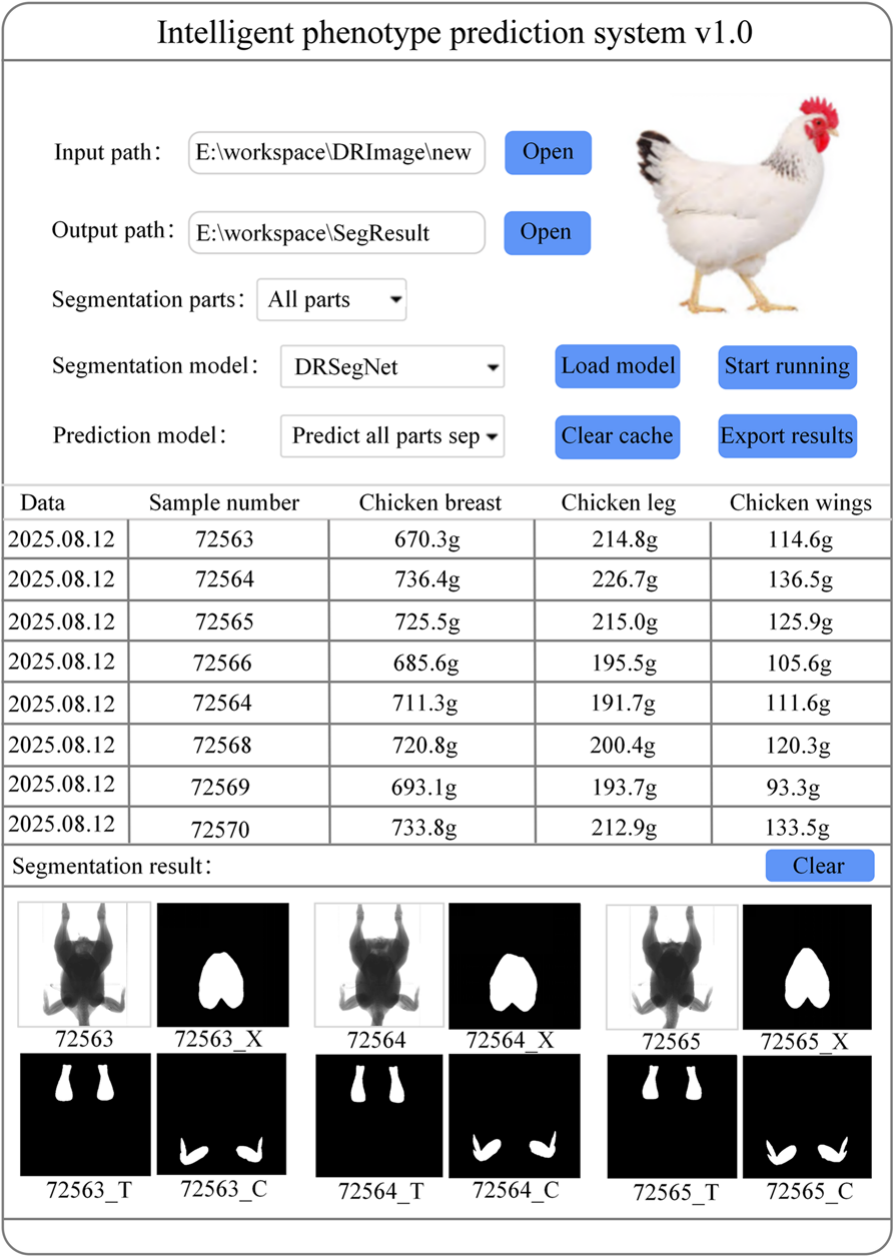
User interface of the integrated phenotypic prediction system. The system enables one-click DR image input and automatically outputs predicted weights of key carcass-related traits, including breast, drumstick, and wing. Segmentation results and prediction outputs are displayed simultaneously, supporting batch processing, result export, and real-time phenotypic evaluation in breeding applications

To support rapid on-site use in breeding programs, an end-to-end phenotyping pipeline was developed. The runtime performance of this pipeline was evaluated to determine its suitability for practical breeding conditions. The full workflow includes image acquisition, preprocessing, segmentation, and carcass trait prediction, and all steps are executed automatically (Fig. 7).

Under real operating conditions, the total time required to obtain predicted breast, drumstick, and wing weights from a single live chicken was kept within 40 s. This time covers all automated computational steps after image acquisition. The results indicate that the pipeline can meet the speed requirements of high-throughput phenotyping.

Table 5 provides a detailed list of the processing time under practical operating conditions. Among them, manual operations such as fixing broiler chickens, positioning them in the chamber, and live-weight measurement accounted for a substantial portion of the total cycle time. In contrast, automated steps including image preprocessing, segmentation, and phenotype prediction take relatively less time, with each sample requiring only about 20 s. The calculation of these time consumption values is based on the average running time calculated from more than 50 images.

**Table 5:** Time consumption of each step in the end-to-end phenotypic prediction workflow

No.	Step	Time
1	Image acquisition	around 8 s
2	Live weight measurement	around 10 s
3	Image processing	around 6 s
4	Image segmentation	6 s
5	Feature extraction and weight prediction	8 s
6	Total processing time	< 40 s

Time values represent the average duration per sample under practical operating conditions. The image processing, segmentation, and prediction steps were calculated based on more than 50 samples. Manual live weight measurement accounted for the largest proportion of total processing time, while the automated components of the workflow were completed within seconds

Overall, the above results indicate that the proposed method can perform automated, near real-time phenotype evaluation of key carcass traits in live chickens, supporting its application in large-scale and routine phenotype measurement.

## Discussion

For a long time, achieving non-destructive measurement of carcass traits such as breast, drumstick, and wing weights has been a key objective in poultry breeding. Traditionally, these traits can only be measured through slaughter, leading breeding programs to rely primarily on sibling testing. This method is not only time-consuming and labor-intensive but also introduces subjective biases from different operators. Although several studies have explored imaging-based phenotyping methods in livestock, each approach has notable limitations. For example, the 2D external imaging methods, such as RGB cameras, can only capture surface features and fail to reflect internal anatomical structures [39]. On the other hand, while CT and MRI provide more detailed anatomical information [40, 41], their imaging acquisition process involves long scanning times, making them difficult to apply in large-scale real-time breeding programs.

To address these challenges, this study proposes a DR imaging-based non-destructive phenotypic prediction framework for real-time estimation of multiple key carcass traits in live chickens. By constructing an image acquisition platform and integrating a multi-objective segmentation model (DRSegNet) with machine learning regression algorithms, intelligent high-throughput phenotyping is realized. The strong consistency between predicted and manually measured values (Table 4) demonstrates that DR imaging can effectively capture valid phenotypic features, providing a reliable foundation for quantitative carcass trait prediction. To further validate the practical breeding applicability of DR-predicted carcass traits, we systematically evaluated the phenotypic and genetic correlations between measured and predicted phenotypes. The results show that the predicted carcass traits can well retain the overall correlation structure of real phenotypic data. Detailed correlation statistics are presented in Table S1.

Compared with traditional 2D surface imaging methods for weight measurement, the DR-based framework can capture internal anatomical structures, thereby providing richer feature information. Meanwhile, in contrast to CT and MRI, DR imaging features simpler operation and relatively faster detection speed, making it more suitable for large-scale breeding applications. The entire workflow from image acquisition to trait prediction takes only approximately 40 s per sample, enabling efficient and standardized high-throughput phenotyping. When combined with automated application software, the framework further enhances operational performance, allowing it to be integrated into routine breeding workflows with limited additional cost and labor.

To translate these prediction results into practical breeding decisions, this study also introduces two quantitative indicators: the PI and the CBI. PI is calculated as the weighted sum of Z-scores—standardized trait values—reflecting the overall phenotypic performance of each individual. In contrast, CBI is defined as the standard deviation of standardized trait values, used to describe the balance between different carcass traits. By incorporating CBI, the risk of producing individuals with imbalanced carcass proportions under single-trait selection can be effectively reduced. Together, PI and CBI provide a more comprehensive and multi-dimensional evaluation reference for identifying ideal breeding candidates.

Unlike traditional sibling testing methods, the DR-based phenotyping approach enables in vivo phenotypic measurement, allowing direct use of the phenotypic information of candidate individuals themselves. This may improve selection accuracy and additionally enables targeted real-time monitoring of their growth status. When combined with the PI and CBI indices, the method further optimizes the breeding process by supporting the transition from single-trait selection to a balanced selection strategy that emphasizes overall carcass yield. Additionally, the high-quality phenotypic data generated by this framework can be effectively integrated into phenotypic and genomic selection workflows, advancing the development of phenomics.

Despite these advantages, this study still has certain limitations. Firstly, as a projection-based technology, DR imaging cannot provide complete 3D anatomical information, which may limit the accurate characterization of some complex internal structures. Secondly, although a standardized protocol was adopted to minimize technical variation, the study has a limitation in that no technical replicates were conducted. Technical replicates could have been implemented by measuring each chicken twice, either at different time points on the same day or with a one-day interval, which would have helped quantify the built-in sources of error inherent in the imaging procedure and improve the reliability of the results. Thirdly, operator bias is inherent in the analysis, which is hidden due to the lack of repeated annotations specifically designed to investigate operator bias and accuracy-this constitutes another limitation of the study. Fourthly, the ground truth values of breast, drumstick, and wing weights were obtained through manual dissection and weighing, which inevitably involve subtle measurement errors despite strict cross-verification by multiple experienced technicians. Such errors are difficult to fully eliminate and may impose an upper limit on the theoretical prediction accuracy of the model. Therefore, future studies will aim to expand validation across multiple poultry populations and species, enhance automation by integrating intelligent image acquisition and data management, and compensate for the lack of complete 3D information by combining DR imaging with supplementary data sources. In parallel, to reduce errors and bias, technical replicates will be performed to quantify inherent technical variation, repeated annotation strategies will be implemented to assess and mitigate operator bias, and more objective, high-precision ground truth systems—such as 3D CT-based volumetric phenotyping—will be established to minimize manual measurement errors. In addition, expanding the framework to cover more carcass related or physiological traits will further enhance its application value in breeding and production environments.

## Conclusion

This study established a non-destructive detection and evaluation framework for the carcass traits of live poultry based on DR imaging and artificial intelligence. Through deep learning image segmentation and machine learning prediction models, live-bird estimations of the weights of the chicken's breast, drumsticks, and wings were achieved, converting post-slaughter traits into measurable phenotypes at the individual level. Additionally, to support comprehensive breeding of multiple traits, the study further constructed PI and CBI. PI quantifies the overall advantages of multiple carcass parts, while CBI assesses their proportional coordination. The combination of the two can help screen individuals with strong comprehensive performance and balanced carcass structure, addressing the limitations of traditional single-trait selection that is prone to causing imbalance in proportions.

In conclusion, this study provides efficient, objective, and multi-dimensional phenotypic measurement and selection tools for poultry breeding, helping to enhance the intelligence level and overall efficiency of breeding decisions.

## Supplementary Information

Additional file 1: Table S1. Phenotypic correlations among measured and predicted carcass traits, and genetic correlations between measured and predicted traits.

Additional file 2: Fig. S1. Performance comparison of six regression models for predicting broiler breast, drumstick, and wing weights. Pairwise Wilcoxon test of prediction errors for six models; Median absolute error bar plot; Boxplots of absolute prediction error; Quantitative performance metrics including Pearson correlation coefficient, root mean square error, mean absolute error, and mean relative error.

## Data Availability

The raw data surveys that support the findings of this study are available from Fujian Sunnzer Biotechnology Development Co., Ltd., but restrictions apply to the availability of these data, which were used under licence for the current study and so are not publicly available. The data are, however, available upon request and with the permission of Fujian Sunnzer Biotechnology Development Co., Ltd.

## Abbreviations

2D: Two-dimensional
3D: Three-dimensional
BN: Batch normalization
CBI: Carcass balance index
CNN: Convolutional neural network
CT: Computed tomography
DR: Digital radiography
ELA: Efficient local attention
GAP: Global average pooling
GCLA: Global context and local attention
GN: Group normalization
GT: Ground truth
IoU: Intersection over Union
LN: Layer normalization
MAE: Mean absolute error
MHSA: Multi-head self-attention
MLP: Multi-layer perceptron
MRE: Mean relative error
MRI: Magnetic resonance imaging
MSFE: Multi-scale feature extraction
PCC: Pearson correlation coefficient
PI: Phenotypic index
RMSE: Root mean square error
SD: Standard deviation
SE: Squeeze-and-excitation
